# A PostgreSQL Tripal solution for large-scale genotypic and phenotypic data

**DOI:** 10.1093/database/baab051

**Published:** 2021-08-14

**Authors:** Lacey-Anne Sanderson, Carolyn T Caron, Reynold L Tan, Kirstin E Bett

**Affiliations:** Department of Plant Sciences, University of Saskatchewan, 51 Campus Drive, Saskatoon SK S7N 5A8, Canada; Department of Plant Sciences, University of Saskatchewan, 51 Campus Drive, Saskatoon SK S7N 5A8, Canada; Department of Plant Sciences, University of Saskatchewan, 51 Campus Drive, Saskatoon SK S7N 5A8, Canada; Department of Plant Sciences, University of Saskatchewan, 51 Campus Drive, Saskatoon SK S7N 5A8, Canada

## Abstract

Researchers are seeking cost-effective solutions for management and analysis of large-scale genotypic and phenotypic data. Open-source software is uniquely positioned to fill this need through user-focused, crowd-sourced development. Tripal, an open-source toolkit for developing biological data web portals, uses the GMOD Chado database schema to achieve flexible, ontology-driven storage in PostgreSQL. Tripal also aids research-focused web portals in providing data according to findable, accessible, interoperable, reusable (FAIR) principles. We describe here a fully relational PostgreSQL solution to handle large-scale genotypic and phenotypic data that is implemented as a collection of freely available, open-source modules. These Tripal extension modules provide a holistic approach for importing, storage, display and analysis within a relational database schema. Furthermore, they embody the Tripal approach to FAIR data by providing multiple search tools and ensuring metadata is fully described and interoperable. Our solution focuses on data integrity, as well as optimizing performance to provide a fully functional system that is currently being used in the production of Tripal portals for crop species. We fully describe the implementation of our solution and discuss why a PostgreSQL-powered web portal provides an efficient environment for researcher-driven genotypic and phenotypic data analysis.

## Introduction

The critical relationship between genotype and phenotype has been understood since the birth of the field of genetics. The advent of genome-wide association studies (GWAS) has allowed researchers to build entire catalogues of single nucleotide polymorphisms (SNPs) that can be associated with various traits of interest. The deluge of genotypic and phenotypic data that researchers currently have to deal with demands a single ‘data warehouse’ for fast, efficient analysis and storage. There have been multiple efforts to successfully manage large data systems (1–6), but many are at the expense of normalization of data or scalability. In particular, Morales *et al.* (6) propose an open-source PostgreSQL solution that links relational phenotypic data with non-relational genotypic data in a single database. However, a limitation of their solution is lengthy query times if a particular query has not been executed before. We have been unable to find an optimal open-source solution that relies primarily on the relational database model for both storage and querying of large-scale biological datasets generated from GWAS, without compromising either performance or data integrity.

Unlike genotypic data, phenotypic data that are captured by individual data collectors can be diverse in terms of assigning values and preferred data formats. While we may be tempted to constrain data collection to ensure consistency of format and use of ontologies, this could result in less accurate trait descriptions and methodology. Therefore, a balance between flexible data input, maximum metadata collection and consistent formats is needed so data can be reusable in the future. Methods for recording phenotypic data collection are continually evolving, ranging from a physical notebook to web applications on a phone or tablet. In the case of plant breeding, one option is Field Book (7), an open-source tablet-based application for recording data while in the field, offering rapid data collection and increased data integrity in comparison to transcribing paper notebooks for a database. Field Book exports data using the specification outlined by BrAPI (8), which standardizes the exchange of breeding data between databases and applications. Ideally, this synergy between BrAPI and Field Book would provide seamless import of phenotypic data; however, collecting phenotypic data through web apps is not yet universally adopted. Incorporation of such technologies into the data collection workflow is also slow due to the learning curve and unwillingness to lose important features already provided by spreadsheet software. Furthermore, most individuals in academia are highly familiar with spreadsheet software and utilizing it as a data-recording tool integrates well with downstream analysis. It also provides great flexibility, such as highlighting important data points or defining methods or scales alongside the data. For example, during a typical data collector workflow, one might wish to mark a measurement to double check later; spreadsheets facilitate this, whereas some web applications may not. Fortunately, most applications can be exported into tabular text formats similar to those exported by spreadsheet software. This means that database systems can be designed to support multiple methods of data collection by focusing on the similarities between tabular text formats, and research groups can opt for a hybrid model of data collection or transition as a whole between methods.

In addition to providing a fast, scalable and secure option for managing large biological datasets, it is also essential to take into account user requirements and skill sets with respect to computational data analysis. While some researchers will self-educate in the realm of command-line interfacing and shell scripting, others feel that it compounds the scientific workload they are already undertaking. In addition, many do not have access to a high-performance computation cluster, causing them to resort to running analyses on their public or private workstations which can tie up their system’s resources for days. Some of these challenges can be overcome by providing a friendly, intuitive interface to analysis tools and searches. A well-designed Graphical User Interface (GUI) can drastically reduce a user’s learning curve and provide a sense of familiarity within their operating system’s environment (9). If the user is expected to install the software, however, they may still run into the issue of having limited resources available on their workstation. GUI software is comparatively slower to command-line analyses due to a greater demand on disk and memory. Additionally, install-only software requires the user to diligently monitor for the latest updates to capitalize on general improvements and new features.

To counteract the limitations of a user’s own computer hardware on storage and computation, a developer can opt to use the web to serve a friendly and dynamic client-side interface with the backend of a dedicated server. Websites can be accessed from anywhere with an internet connection by anyone, with the potential to password-protect some or all content. Use of the web lends itself to accessibility, portability, manipulation and analysis of data through searches, export and integration of metadata, all with simple access to the internet and a browser. This can facilitate meeting findable, accessible, interoperable and reusable (FAIR) data principles (10) and thus becomes a highly advantageous option to gain the greatest potential from both the data itself and the researchers that do the analyses.

The use of a content management system, such as Drupal (11), further benefits the developer by allowing them to shift focus from the overhead of managing users and website security, to the content and user experience. Tripal (12) extends Drupal with added functionality for biology-based web portals including specialized displays, data management tools and data importers. Tripal stores all biological data in the GMOD Chado schema (13) which has a focus on ontology-driven metadata and generic data categories (i.e. features, stocks and analyses) with rich linking tables supporting flexible interconnectivity of data. This generic, modular schema includes the Natural Diversity Module (14) to provide flexible support for genotypic, phenotypic and field collections. As described in Jung *et al.* (14), the Chado Natural Diversity module requires 10 tables to describe a genotypic data point with multiple records per table to describe important metadata. This becomes a serious limitation in the case of large genotypic datasets as the number of records involved degrades performance through multiple table joins. Morales *et al.* (6) uses the Chado Natural Diversity tables as a relational link to genotypic data primarily stored non-relationally within the same database. While this improvement provides concrete links between genotypic and phenotypic data and reduces the number of records needed, it still requires the same number of table joins to fully describe the data and does not take advantage of the ontology-driven nature of Chado for metadata storage.

Chado is designed to use PostgreSQL (15), an open-source relational database model that is rich in features and also stable and reliable as a result of being atomicity, consistency, isolation and durability (ACID)-compliant. ACID compliance ensures that, if all principles are met, data housed in a database will retain validity regardless of whether multiple transactions are occurring at once or if there is a power failure or disruption (16). Chado uses constraints within PostgreSQL to ensure data types, relationships and boundaries are respected. However, relational databases are known to be demanding on storage requirements and difficult to maintain without comprehensive knowledge of the database structure (3). Complex schema such as Chado with many interrelated tables can require extremely long queries to extract datasets from; however, this can be mitigated through the development of targeted, efficient indices. Traditionally, genotypic data are represented in matrix format and many solutions attempt to mimic this format in PostgreSQL (1–6, 17). While the flexibility of array- or JSON-based data types in PostgreSQL provides an intuitive way to store raw data points, using these as the core of a storage model has the consequence of moving outside the data constraints and ontology enforcement in Chado. Thus, to extend Chado for efficient storage of genotypic data with strong data integrity, it is important to extend the current relational principles for core data where non-relational data types play a secondary role as appropriate. Here, we investigate and propose an all-encompassing efficient storage, data loading and access solution for genotypic and phenotypic data in biological web portals using Tripal and the Chado schema. Optimization of indices and minor adjustments to the existing Chado data model has enabled us to provide a performant and scalable option for dozens of web portals that are a part of the Tripal community.

## Implementation

The functionality described here has been packaged as three Tripal modules: ND Genotypes (https://git.io/fpH7L), Genotypes Loader (https://git.io/fpH7Y) and Analyzed Phenotypes (https://git.io/fpH7l). Deployment is on a Tripal instance with data stored exclusively in PostgreSQL. Data are accessed through the Tripal web interface in the form of searches, tools and integration on genetic marker, variant and germplasm pages. These modules are tightly integrated with Drupal permissions to provide secure access control.

## Data model

**Figure 1.**
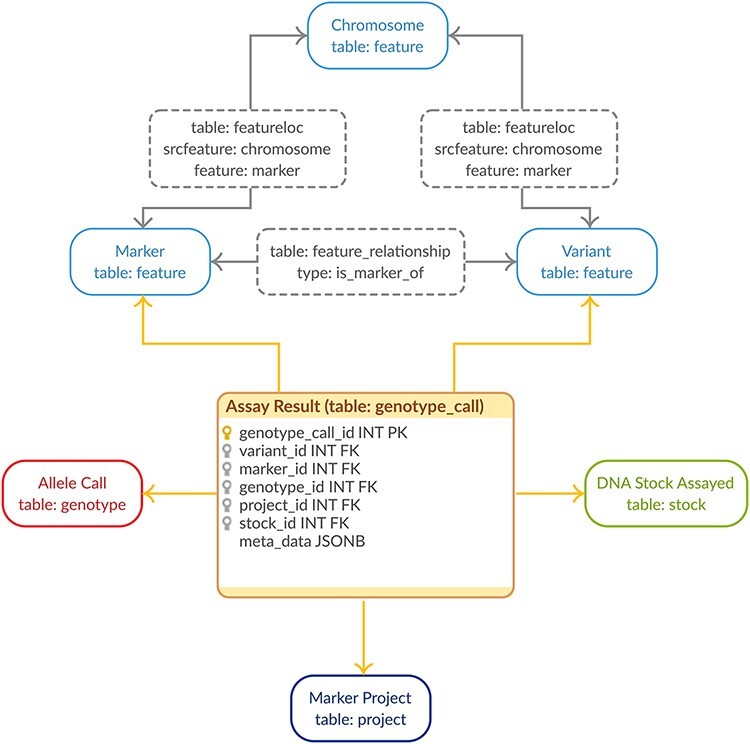
Genotypic Data Model. The entity–relationship diagram describes the relevant sections of the Chado schema for storage of genotypic data. The genotype_call table (highlighted in orange) is a backward-compatible enhancement to the Chado schema, whereas all the supporting tables match the generic Chado specification. The genotype_call table gathers together all the metadata for a genotypic data point into a single record through the use of foreign keys.

Genotypic data are stored in PostgreSQL using the GMOD Chado schema (13) with minor, backward-compatible improvements. All metadata documenting projects, genetic markers, sequence variants and germplasm data are stored in their respective unaltered Chado data tables. The genotypic calls are stored in a new linker table joining the project, markers, variants and germplasm with the allele call (Figure 1). This method of gathering all the relevant relational pieces into a single record drastically cuts down on storage space while the use of foreign keys ensures referential integrity (18). Using a linking table fits well with the Chado model while using at the same time uses fewer tables than the Chado Natural Diversity module leading to a more compact storage solution specific to genotypic data. The genotype_call table also contains a JSONB metadata column for efficient, flexible storage of genotype properties (e.g. quality metrics). This flexibility allows us to store all genotype properties supplied to reflect the Variant Call Format (VCF) (19) specification which allows complete flexibility in genotype properties available. The allele call indicated in the genotype field is translated to nucleotides and stored in the chado genotype table to ensure the call is fully specified within the relational schema. In this way, we take advantage of modern PostgreSQL (i.e. version 9.4+) to store the metadata available for our genotypic data efficiently and flexibly while still keeping the core genotypic data normalized. This is in direct contrast to existing solutions for high-density genotypic data (1–6).

**Figure 2.**
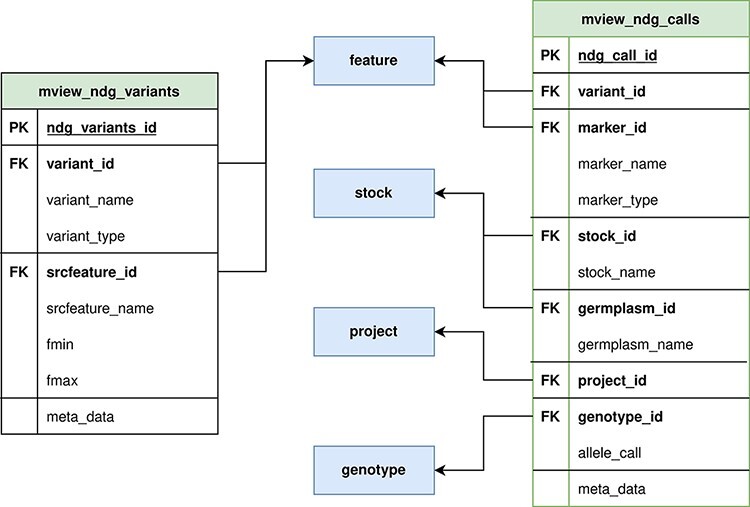
ND Genotypes materialized view implementation. Mview_ndg_variants (left) contains all variant locations providing support for multiple genome assemblies without duplicating the allele data stored in mview_ndg_calls (right). Both these materialized views pull data from several Chado tables (center, blue) to prevent table joins in user-triggered queries. There are numerous foreign keys in these materialized views to allow retrieval of infrequently required metadata.

Our approach prioritizes data integrity and full metadata preservation; however, as a web client application, query speed is also critical. With this in mind, we created two generic materialized views (Figure 2) with the intent of minimizing query JOINS, while still supporting very different queries with a single materialized view. This approach allows us to optimize query speed and storage space at the same time, compared to the single materialized view per query approach. The main materialized view, mview_ndg_calls, compiles display names for the data linked in the genotype_call table (i.e. project, genetic marker and stock). This materialized view supports both variant and germplasm-focused queries. Mview_ndg_variants compile variant locations allowing support of multiple locations per variant (e.g. multiple genome assemblies) without increasing the magnitude of the materialized view for genotype calls. Well-chosen indices (Supplementary Data 1) were designed to improve query speed while optimizing storage space.

**Figure 3.**
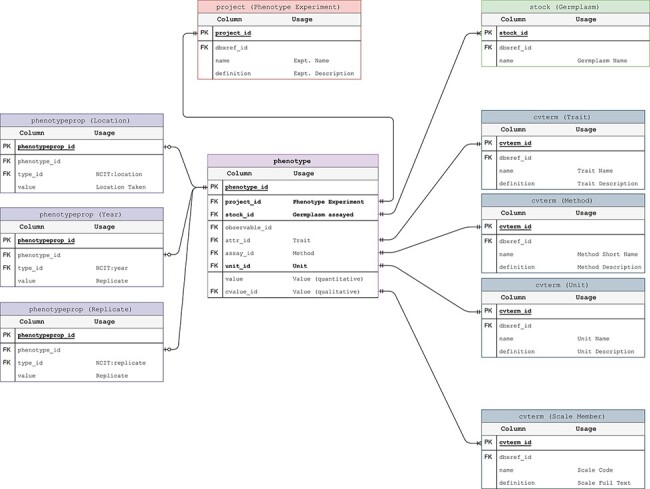
Phenotypic Data Model. The entity–relationship diagram describes the relevant sections of the Chado schema for storage of phenotypic data. This data model adheres to the standard Chado schema with the addition of stock, project and unit foreign keys to the phenotype table. The change is backward compatible but critical in that it allows us to store the trait-method-unit tuple in the phenotype table for each phenotypic data point.

Phenotypic data are also stored in the GMOD Chado schema with trait data stored as controlled vocabularies (i.e. in the cvterm table) and measurements stored in the phenotype table. The germplasm measured is the same accession stored in the stock table for genotypes (Figure 3). This ensures the connection between phenotypic and genotypic data is preserved and unambiguous. The only departure from the published Chado schema is the addition of a stock_id, unit_id and project_id to the phenotype table. This allows for more efficient linking of measurements to their component parts thus minimizing space requirements and improving performance.

As with genotypes, phenotype query performance is also optimized using generic materialized views. There is a single materialized view, mview_phenotype, which averages across replicates and compiles display names for the linked data (i.e. trait, method, unit, project and site-year). This single materialized view is flexible enough to provide data for all queries required by the module and improves performance while still preserving the original data for novel research. Query speeds were optimized through strategic indices (Supplementary Data 1).

## Data loading

Fast loading of large datasets in the VCF is provided by the Genotypes Loader module, which supports VCF v4.0+. Administrators are given the flexibility to configure controlled vocabulary terms which describe the data in the context of their web portal. Additionally, the loader will always check for pre-existing germplasm and variants and can be configured to either enter germplasm and variants from the VCF directly into the database or fail with an error message if they do not yet exist. The loader validates the VCF to ensure that it matches the specification and extensively checks Chado constraints to provide descriptive error reporting to the user. PostgreSQL’s COPY FROM command is used for inserting all metrics within the genotype fields of the VCF into the genotype_call table. This ensures that multiple genotype calls, the number of which is configurable, are processed within a single database query versus one query per genotype call, thereby drastically improving the speed of import while ensuring constraints are met. Missing genotype calls in the VCF file are skipped during import to optimize storage space and query speeds, which is particularly impactful in the case of sparse datasets (17).

**Figure 4.**
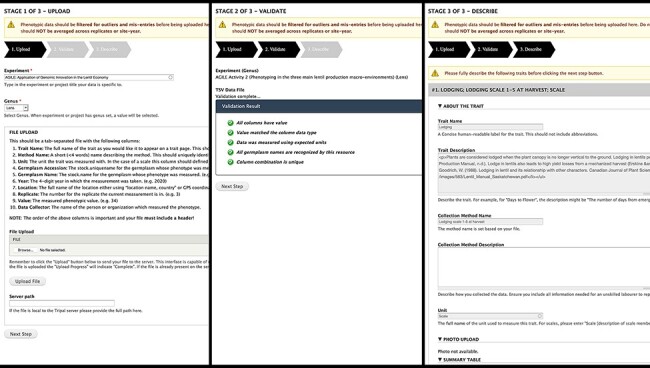
Analyzed Phenotypes Upload form. The left panel shows the first stage of upload, where the user selects their experiment and genus and uploads their file in TSV format. Stage 2 (middle) processes the file and reveals green checkmarks where validation has passed and red *x*’s with helpful guidance if there were errors. The user can re-upload their file if there are issues or move onto the final stage (right) where they will be asked to describe each trait in their file. If a trait name matches exactly to an existing trait in the database, the fields will be gray and cannot be changed as seen in the screenshot but provide a valuable resource for the user to validate that the methods and units used are the same.

We opted for a GUI for phenotypic data upload (Figure 4). Data are uploaded in TSV format, which is easily exportable by spreadsheet software; furthermore, data collection apps like Field Book can also export in easily transformed column-based formats. Our template specifies core metadata such as trait, method, unit and germplasm accession with each row representing a single measurement. Validation occurs instantly with descriptive feedback to guide the researcher through formatting errors or valuable missing information. The entire file undergoes data-level validation to ensure data types match expectations, related data already exist and all required data are supplied. Due to the differences of phenotypic data methodology between researchers, having each researcher upload and validate their own data improves data quality by limiting transcribing mistakes and ensuring they are describing their own methods and units. Researchers are also asked to map their traits to standardized ontologies to facilitate data sharing. Access to the data upload is configured by the site administrator and may be restricted to specific users or more generally to users with a given role.

## Display

**Figure 5.**
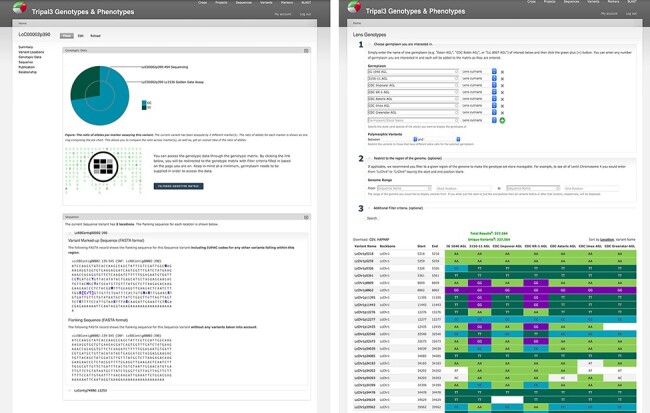
Genotypic data visualization on a generic Tripal site. The left panel shows the distribution of alleles for the current variant as a pie chart (top), a link to the genotype matrix (middle) and the flanking sequence for the variant with known additional variants indicated by their IUPAC codes (bottom). The right panel shows the genotype matrix tool with the allele consensus for seven user-selected germplasm displayed in a variant by germplasm accession table. This table can be further filtered by pairwise polymorphism, genomic region as well as additional filter criteria.

There are several ways to access genotypic data provided by the system. Tripal provides genetic marker and sequence variant pages supporting metadata such as location, primers and assay type; our ND Genotypes module provides Tripal Fields to summarize genotypic data on these pages. Pie charts show the ratio of observed alleles for a given marker to provide context to a researcher’s marker assay results. These charts are generated on demand from the data storage described previously through specialized web services and D3.js is used to draw SVG diagrams supporting user interaction. A separate field provides the flanking sequence of the marker or variant with known variants indicated (Figure 5) by their International Union of Pure and Applied Chemistry (IUPAC) codes (20). This is particularly useful for marker assay design to take advantage of new technologies. Both of these Tripal fields provide configuration options following Tripal best practices and, as such, can be repositioned on the page through the Tripal Display Suite.

ND Genotypes provides a variant by germplasm genotype matrix tool that facilitates the quick lookup of genotypes for a specific germplasm set and/or genomic region (Figure 5). This matrix displays the nucleotides to the researcher with empty cells in the table to represent missing data points. Powerful filtering options such as metadata and polymorphism between a pair of germplasm accessions allow researchers to restrict large amounts of data into high-quality datasets suitable for analyses. Dynamic queries are used to first retrieve the set of variants matching filter criteria from the variant-focused materialized view and then, in a separate query, retrieve the genotypic calls for those variants from the allele-focused materialized view. This approach improves performance by filtering the much smaller variant materialized view and ensures we can use the primary key index for fast retrieval from the larger genotypic call materialized view. Download is available in common formats such as CSV genotype matrices and Hapmap to facilitate external analysis.

**Figure 6.**
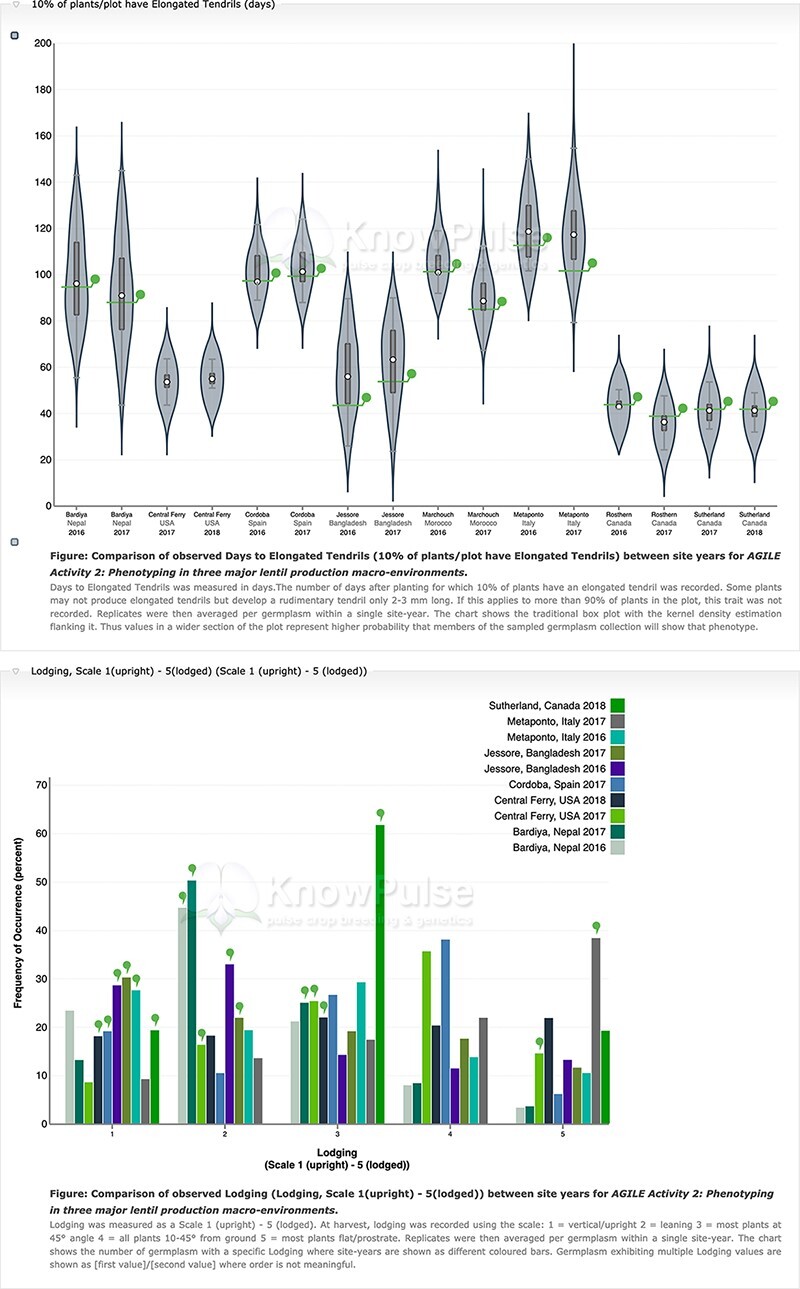
Phenotypic data visualization on a generic Tripal site. Both visualizations are available on specific germplasm pages and show the distribution of phenotypic data collected separated by site-year with the current germplasm indicated by a green indicator. Violin plots (top panel) are used to visualize quantitative data with the mean value shown on the *y*-axis and the site-year indicated along the *x*-axis. Histograms (bottom panel) are used to visualize qualitative data with the frequency of occurrence on the *y*-axis, the scale shown along the *x*-axis and each site-year indicated by bar color.

Our Analyzed Phenotypes module summarizes phenotypic data by trait in individual distribution plots where replicates are averaged but site-years remain separate. Violin plots are used for quantitative data with box plots to show both data structure (i.e. median, interquartile range and 95% confidence interval) and distribution (Figure 6). Qualitative phenotypic data are summarized using histograms (Figure 6). Both charts are data-driven using specialized web services, interactive using d3.js and enable comparison among site-years. On germplasm pages, individual germplasm of interest are highlighted. Trait distribution plots can be accessed through a single page application that generates them based on user input and through specialized Tripal Fields provided by our module for Tripal trait, germplasm and experiment pages. This kind of integration between data and the rest of the Tripal system ensures that the display is intuitive to all users by providing context and summaries for the trait, experiment or germplasm being viewed.

## Demonstration

**Figure 7.**
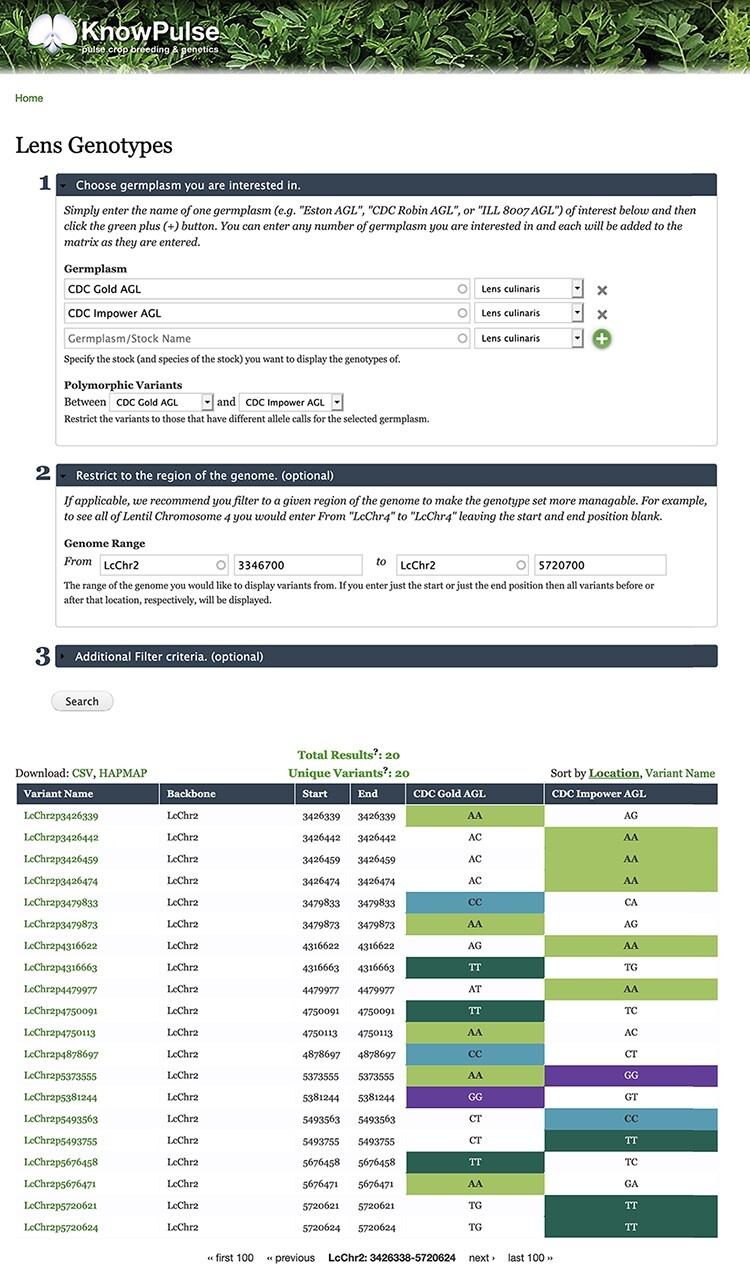
Lentil Genotype Matrix functionality provided by ND Genotypes module. This screenshot shows the listing of polymorphic genotypic data for CDC Gold AGL and CDC Impower AGL restricted to LcChr2:3346700..5720700. This pre-filtered view can be accessed at https://knowpulse.usask.ca/AGL-Lc1.2-Matrix-Example. Alternatively, the user can access the genotype matrix tool and enter the filter criteria as shown in the screenshot starting with the germplasm names.

The Tripal modules described above are a core part of KnowPulse (21), a genetic, genomic and phenotypic web portal for pulse crops. Sequence variant information for a diversity panel of lentil (*Lens culinaris*) (22) in VCF has been loaded into KnowPulse using the Genotypes Loader module. To demonstrate some of the functionality of the ND Genotypes module, consider the following example question: given a region in the genome shown to contribute to a trait, which variants are polymorphic between two germplasm accessions? The researcher can navigate to the form for Lens Genotypes on KnowPulse (https://knowpulse.usask.ca/gmtrxLc). Figure 7 reflects the resulting tabular view from specifying two germplasm lines (CDC Gold AGL and CDC Impower AGL) and selecting each line from the dropdown for polymorphic variants in the first portion of the form. The dataset was further restricted by specifying a range 3 346 700–5 720 700 on chromosome LcChr2 in the second portion of the form. The researcher now has a list of variants that are polymorphic and can page through their results or download in CSV or Hapmap format (if they are logged into KnowPulse with the appropriate permissions).

**Figure 8.**
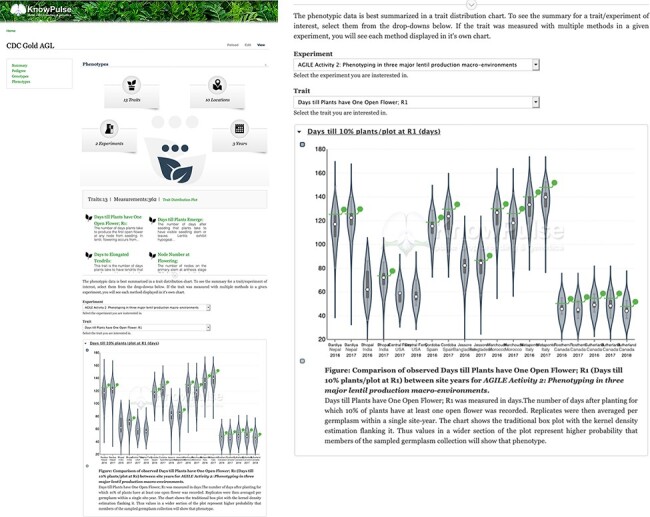
Phenotypic data visualization for CDC Gold AGL provided by Analyzed Phenotypes. The screenshot on the left shows the phenotypic data pane open on the KnowPulse germplasm page for CDC Gold AGL. The top section summarizes the phenotypic data available for CDC Gold AGL, the middle section provides quick links to all traits with data for CDC Gold AGL and the bottom section embeds the ‘Days till 10% of Flowers have one open flower’ Trait Distribution plot for this germplasm. The user selects the trait and experiment combination, and the plot is dynamically drawn for them. The screenshot on the right magnifies the trait distribution plot. This page can be accessed at https://knowpulse.usask.ca/germGoldAgl or by searching for CDC Gold AGL using the Germplasm search tool.

To demonstrate AnalyzedPhenotype functionality, let us assume a researcher is interested in flowering traits for specific germplasm with the goal of comparing it to a germplasm diversity panel. By searching for their germplasm of interest (https://knowpulse.usask.ca/germGoldAgl) and clicking on or scrolling to the ‘Phenotypes’ field, they can specify their experiment (in this case, AGILE 2) and trait of interest (Days till Plants have One Open Flower) in the dropdowns provided. Figure 8 shows the distribution of flowering time for the given experiment. The germplasm of interest is highlighted using a green line bisecting each violin which indicates the value in each site-year relative to the other germplasm in the experiment. In this example, we see that CDC Gold AGL typically flowered later than average in most site-years.

## System and methods

All the extension modules described in this article were developed to support large genotypic and phenotypic datasets for intuitive, quick exploration by researchers and metadata-rich distribution. They were developed to be modular and highly configurable to facilitate sharing among the Tripal community.

### Code standards and accessibility

All modules are open-source (GPLv3) and freely available on GitHub: ND Genotypes (https://git.io/fpH7L), Genotypes Loader (https://git.io/fpH7Y) and Analyzed Phenotypes (https://git.io/fpH7l). Each module features extensive documentation hosted by ReadtheDocs including installation, usage and guidelines for collaboration. Furthermore, care has been taken to ensure these modules meet the standards of the Tripal community as defined by the Tripal Rating 2019 System (23), which ensures that they can be used on a generic Tripal site. Specifically, all three modules have a gold badge indicating the highest level of standards recognized by the Tripal community. These modules will be upgraded to the next version of Tripal when it is released and there is strong intent to maintain them. We encourage questions, suggestions or bugs to be posted in the associated issue queue.

### Benchmarking

Benchmarking was done on both simulated and real-world datasets in a production Tripal website with a small and uneven load. The tests were run nine times on the same day over the span of at least 4 h to help mitigate the differences in load. Queries were timed at the database level using PostgreSQL 9.4.10 EXPLAIN ANALYZE and as such does not include rendering time in Tripal. This uses the PostgreSQL query planning functionality while the addition of the analyze keyword ensures the query is actually run and the total execution time is reported. Each dataset exists within a production database. The datasets for the benchmarking described in this article were hosted on a Linux database and webserver combination. The database server is a Lenovo X3650 M5 with 2× Xeon 6C E52643 V3 3.4 GHz processor, 128 GB TruDDR4 RAM, 8 × 600GB 15K 6 Gbps SAS 2.5in G3HS HDD in a RAID10 configuration.

Real-world datasets consist of data generated by the University of Saskatchewan Pulse Crop research group with a focus on the AGILE Genome Canada grant. Specifically, genotypic data consist primarily of the *L. culinaris* exome capture assay (22) and phenotypic data consist primarily of the *L. culinaris* AGILE diversity panel with a focus on phenology traits (24). The genotypic dataset consists of 105 340 269 data points across 534 individuals and 372 506 variants. The phenotypic dataset consists of 39 302 data points across one experiment, 15 traits, 23 site-years and 451 individuals.

Tripal Test Suite (25) database seeders have been included in ND Genotypes (26) and Analyzed Phenotypes (27) with documented instructions for use. These database seeders were designed to approximate real data as closely as possible with the exception that there are no missing data points, since our data importers would exclude these data points. They can be used on any Tripal-compatible system to stress-test genotypic and phenotypic data storage and visualization. This approach ensures prospective adopters can test this solution on their system.

Benchmarking for ND Genotypes features three queries that cover the genotypic matrix in three states—(i) Unfiltered: germplasm selected but no filter criteria; (ii) Range: germplasm selected with a genomic sequence range specified and (iii) Polymorphic: germplasm selected and only polymorphic variants shown. Analyzed Phenotypes benchmarking features two queries—(i) Quantitative Measurement Distribution: the query used to generate the trait distribution plots and (ii) Summary: specifies the magnitude of data per genus (e.g. the number of experiments). Full details of these queries can be found in Supplementary Data 1.

## System requirements

ND Genotypes, Genotypes Loader and Analyzed Phenotypes require a Tripal 3 (12) instance: Apache2 (28), PostgreSQL 9.4+ (15), PHP 5.6+ (29) and Drupal 7.× (11). Hardware requirements depend largely on the magnitude of data stored with the limiting factor being disk space.

## Discussion

### Why a web portal?

As we established in the introduction, there are many hurdles to researcher’s use of existing large data technologies. Since existing implementations are primarily focused on power users with most requiring command-line experience, they are not accessible to many researchers. Furthermore, these implementations may be overkill for more focused questions such as ‘What variants are polymorphic between my germplasm accessions of interest in a trait-implicated region of the genome?’ or ‘Is this germplasm accession early or late flowering compared to the rest of our diversity panel?’. Our web-based implementation provides a means for researchers to explore these questions quickly and intuitively, without the need to install software, requisition time on a high-performance computing cluster, develop analysis scripts or require assistance from a bioinformatician.

An additional benefit of a web-based implementation as compared to command line-based implementations is the ability to summarize and display data through powerful visualizations. For example, our Analyzed Phenotypes module provides data summarized in a violin plot for intuitive comparison between site-years. Thus, in addition to simply providing the data to answer a given question, our modules also generate publication-ready figures without additional software or analysis.

Lastly, through the use of a web portal, these modules provide a way to meaningfully distribute data. Rather than handing a spreadsheet off to a collaborator, researchers can share the URL for the content page (i.e. a trait, experiment or germplasm) allowing collaborators to access not only the data but also critical metadata and visualizations to ensure that data are meaningful. Essentially, the use of Tripal with these extension modules can help your data adhere to FAIR data principles. Tripal already goes a long way toward making your data FAIR (10) and these modules tie into the Tripal application programmers’ interface (API) to ensure the preservation of all the benefits of Tripal. Specifically, our modules utilize the Tripal API to provide: (1) web services and searches to increase data findability, (2) data stored in the common Chado community schema to increase interoperability between data types and projects, and (3) extensive support for metadata documentation to increase data reusability.

### Why a PostgreSQL normalized schema?

When considering storage options for large-scale biological data web portals, data integrity combined with query performance are key considerations. PostgreSQL is a common choice for the storage of biological data (1–6, 30–35), as it has robust data-type checking and a rule-based constraint system meeting ACID compliance. These powerful tools for data integrity have been used extensively in Chado, which ND Genotypes and Analyzed Phenotypes extend.

Within the framework of PostgreSQL, one can implement an array-based technique as described by Lichtenwalter *et al.* (3) or a more traditional relational structure. While array-based implementations provide flexible data definitions, faster queries and more compact storage (3), it is at the expense of constraint checking ensuring referential integrity. Furthermore, we opted for a normalized, relational database approach because it excels at answering diverse questions without data duplication (36). For example, genotypic questions include (i) what is the allele frequency of a particular variant, (ii) how many variants are in a particular region of interest, (iii) how many germplasm accessions have a particular allele and (iv) which variants are polymorphic between a pair of germplasm accessions? All of these questions can be answered using specialized queries acting on a single normalized relational schema, whereas with a non-relational approach the data would need to be duplicated per query to optimize speed (17).

In addition to the data integrity built into Chado, our fully relational solution ensures genotypic and phenotypic data are integrated tightly with other biological data types through existing Chado linking tables. This, combined with Tripal’s support for Chado, allows us to provide an extendable, full web portal solution with a vibrant open-source community. The Tripal API provides Tripal Fields to enable linked data to be displayed on Tripal Content pages. Our modules use this API extensively to display genotypic and phenotypic data summaries on all linked data pages (i.e. germplasm, experiment, marker and variant pages). Furthermore, Tripal’s integration with Drupal Views ensures administrators can make searches across data types, such as restricting germplasm based on genotypic and phenotypic data. This is possible because our data storage solution uses relational links for variants, markers, germplasm, experiments and even alleles. This contrasts with solutions such as BreedBase (6), which do not have relational links to the Chado feature table for markers due to their storage within a JSONB object. Additionally, their storage design stores all markers for a given germplasm experiment combination in a single JSONB object which does result in a limit on the number of markers, whereas our solution only experiences the shared limit to PostgreSQL database size.

## Optimizing performance

Within a normalized relational schema, performance can be optimized through well-placed indices and flexible materialized views. ND Genotypes and Analyzed Phenotypes use both indices and materialized views to optimize query speeds for use within the web environment as described under Implementation.

Chado is a highly normalized database schema that supports a variety of biological data types in a highly interconnected manner. Furthermore, it provides flexible storage for associated metadata through property (e.g. stockprop) and related controlled vocabulary term (e.g. stock_cvterm) tables. However, this flexibility comes at the cost of requiring a large number of table joins to complete most queries which results in longer query times as joins are compute intensive. For performance reasons, materialized views are used to aggregate the data ahead of time into a single question-independent table, which allows user-triggered queries to contain a much smaller number of table joins. For comparison, user-triggered queries in BreedBase (6) are run directly on the Chado Natural Diversity schema requiring multiple joins to link the genotypic data with the experiment and germplasm. While they mitigate this using a file-based cache for repeated questions, if your user base is asking a large variety of questions, then fewer table joins imply our method would be more performant.

ND Genotypes and Analyzed Phenotypes use materialized views to speed up queries but do so in a flexible manner to ensure multiple queries can use the same materialized view. Well-chosen indices were then added to the materialized views to further optimize performance. It is important to remember that not all indices will speed up your queries. Extensive research using the PostgreSQL query planner was done on each of the queries executed by these modules to ensure that (i) the best indices for each query were available and (ii) no additional unused indices were in place taking up storage space. When designing indices where storage space is a concern (e.g. genotypic datasets), the best indices are a balance between query-specific compound indices for optimal query speeds and reusable indices focused on multiple queries. For example, range queries are time consuming, so we used a compound index and segregated variant location information from genotypic calls to restrict a genomic range with a smaller index. However, filtering for pairwise polymorphism using a compound index requires too much storage space; therefore, we opted for reusable indices paired with join subqueries. Our approach also provides hints to the query planner to ensure optimal index use through the query itself by utilizing JOIN subqueries, the EXISTS operator and ROW constructors. Thus, our solution finds a balance between performance and storage space.

**Figure 9.**
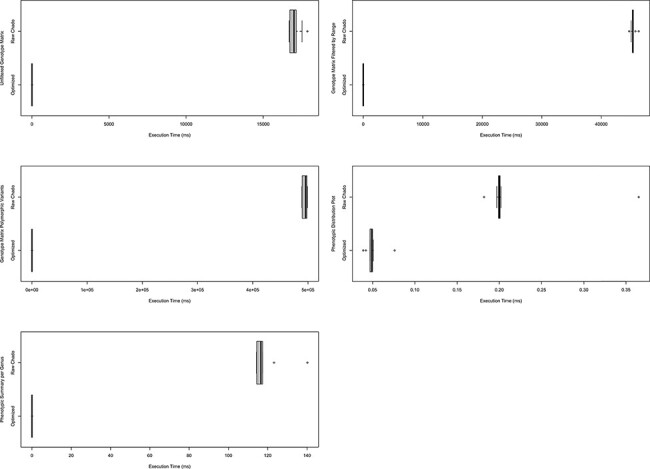
Comparison of timings for functional queries on indexed materialized views versus Chado. The left column shows timings for genotypic data-focused queries and the right focuses on phenotypic data queries. Each box plot represents functionality in either Analyzed Phenotypes or ND Genotypes with the *x*-axis indicating the execution time of the query is milliseconds and the *y*-axis indicating the query performed. All box plots show the optimized form of the query was substantially faster than querying Chado directly. These timings were taken on a production database with nine replicates over the course of 1 day. Queries are fully described in Supplementary Data 1 and methods were described under Systems and Methods: Benchmarking.

ND Genotypes and Analyzed Phenotypes have been used in production settings. As described in the Benchmarking section under System and Methods, the data queries behind existing functionality were timed either using the materialized views with indices or querying Chado directly. We observed a compelling increase in performance as shown in Figure 9. Not only do these performance gains justify our approach, but they also show that this solution works in production on large datasets. Furthermore, both modules include Tripal Test Suite Database Seeders which ensure you can stress test performance on your own system (26, 27).

## Conclusion

To summarize, the collection of modules described in this article provide a performant web-based solution to storage, distribution and analysis of large phenotypic and genotypic datasets. Specifically,

- Materialized views and strategic indices make our data storage method efficient on large datasets. Each module provides researchers with dynamic visualizations, queries and filterable downloads.
- Our web focus makes large datasets accessible to researchers by facilitating exploration without installation of software or typical data acquisition and reformatting.
- As all data including quality meta-data are stored in a relational database with referential integrity, such web portals provide a quality backup of data.
- Multiple experiments can be housed in the same web portal in an integrated and searchable manner facilitating comparison between datasets.
- All data are displayed alongside quality, descriptive metadata with download functionality to ensure datasets are reusable.

Essentially, this collection of modules, in combination with Tripal, provide a solution to make large genotypic and phenotypic datasets FAIR while also providing a framework for researchers to explore the data.

## Supplementary Material

baab051_Supp
