# Supplementary material for: A PostgreSQL Tripal solution for large-scale genotypic and phenotypic data
Source: Database (Oxford). 2021 Aug 14;2021:baab051. doi: 10.1093/database/baab051 (PMC8363843; doi:10.1093/database/baab051)
Supplement: baab051_Supp [file baab051_supp.zip › Sandersonetal.SupplimentaryData1.pdf]

Supplimentary Data 1: Benchmarking

This document describes the queries used for benchmarking in the discussion. Each query is executed by the specified extension module in the described case.

ND Genotypes

The queries tested represent those executed by the genotype matrix provided by this module. Specifically, we focused benchmarking on the genotype matrix tool since the queries executed access the most data.

Unfiltered Query

This is the query executed when you specify a set of germplasm to see genotypes for but do not specify any other criteria. Specifically, the following query represents the case where 10 germplasm have been specified (denoted by :germplasm_0 through :germplasm_9 placeholders in the query).

The following SQL statements represent accessing the data through the optimized materialized view with indices versus through querying the chado tables directly.

Optimized through Materialized View and Indices

```
SELECT v.*, cf.nid
FROM chado.mview_ndg_lens_variants v
LEFT JOIN public.chado_feature cf ON cf.feature_id=v.variant_id
WHERE
  EXISTS (
    SELECT true FROM chado.mview_ndg_lens_calls call
    WHERE germplasm_id IN (:germplasm_0, :germplasm_1, :germplasm_2, :germplasm_3, :germplasm_4, :germpl
      AND call.variant_id=v.variant_id )
ORDER BY srcfeature_name ASC, fmin ASC
LIMIT 100
```

Raw Chado query to match the optimized version above

```
SELECT gc.variant_id, gc.marker_id, m.name as marker_name, mt.value as marker_type,
      gc.stock_id, s.name as stock_name, g.stock_id as germplasm_id, g.name as germplasm_name,
      gc.project_id,  gc.genotype_id,  a.description as allele_call, gc.meta_data, cf.nid
    FROM chado.genotype_call gc
      LEFT JOIN public.chado_feature cf ON cf.feature_id=gc.variant_id
      LEFT JOIN chado.feature m ON m.feature_id=gc.marker_id
      LEFT JOIN chado.featureprop mt ON mt.feature_id=m.feature_id AND mt.type_id=4321
      LEFT JOIN chado.stock s ON s.stock_id=gc.stock_id
      LEFT JOIN chado.organism o ON o.organism_id=s.organism_id
      LEFT JOIN chado.stock_relationship sr ON sr.subject_id=s.stock_id AND sr.type_id=3712
      LEFT JOIN chado.stock g ON g.stock_id=sr.object_id
      LEFT JOIN chado.genotype a ON a.genotype_id=gc.genotype_id
    WHERE g.stock_id IN (:germplasm_0, :germplasm_1, :germplasm_2, :germplasm_3, :germplasm_4, :germpla
```

Both of the above queries were run on a production database in order to benchmark query execution times. This was done using EXPLAIN ANALYZE [query] which ensures the query is actually executed by the

PostgreSQL query planner over the course of a single day with 9 replicates taking at least 4 hours to complete. The data was then plotted below:

```
data.unfiltered = Benchmarking.Genotypes[Benchmarking.Genotypes$query == 'Unfiltered Genotype Matrix', ]
boxplot(data.unfiltered$execution.time~data.unfiltered$query.target,
        horizontal=TRUE, ylab="Unfiltered Genotype Matrix", xlab="Execution Time (ms)")
```

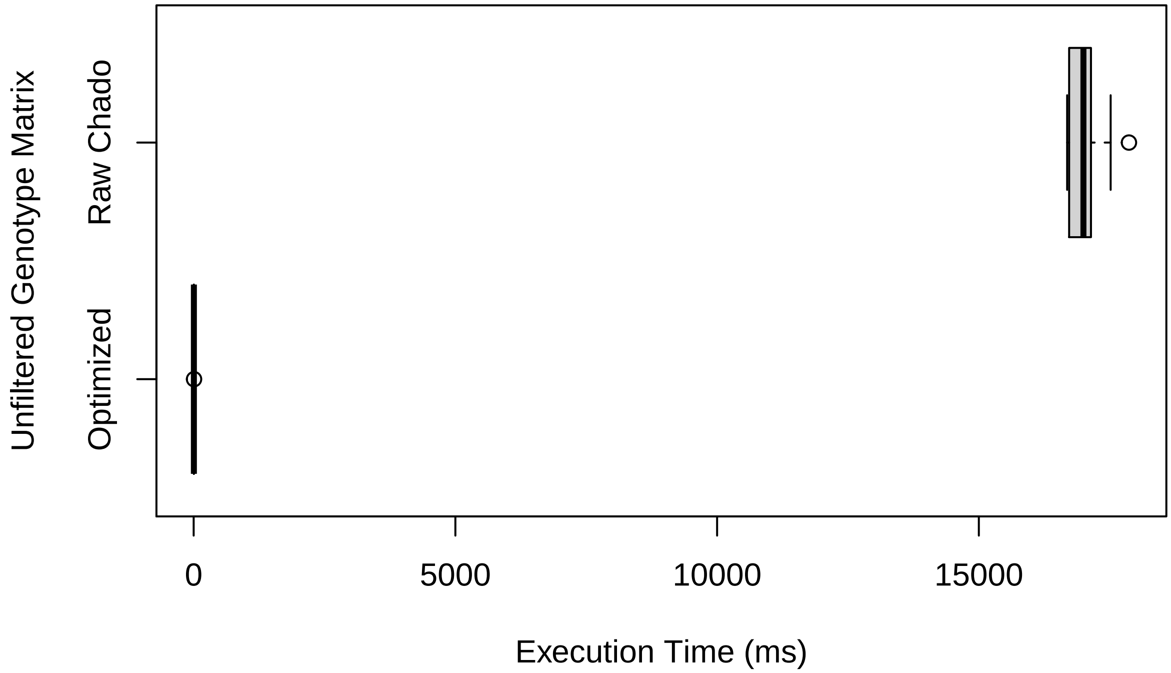

Range Query

This is the query executed when you restrict the set of variants returned to a specific region in the genome. Specifically, the range requested is specified by start chromosome(:sbackbone) and position(:sfmin) to end chromosome(:ebackbone) and position(:efmin) in the query below. Additionally the query includes the same 10 germplasm used for the "Unfiltered Query" (denoted by :germplasm_0 through :germplasm_9 placeholders in the query).

The following SQL statements represent accessing the data through the optimized materialized view with indices versus through querying the chado tables directly.

Optimized through Materialized View and Indices

```
SELECT v.*, cf.nid
FROM chado.mview_ndg_lens_variants v
LEFT JOIN public.chado_feature cf ON cf.feature_id=v.variant_id
WHERE
  ROW(v.srcfeature_name, v.fmin) BETWEEN ROW(:sbackbone, :sfmin) AND ROW(:ebackbone,:efmin)
  AND EXISTS (
    SELECT true FROM chado.mview_ndg_lens_calls call
    WHERE germplasm_id IN (:germplasm_0, :germplasm_1, :germplasm_2, :germplasm_3, :germplasm_4, :germpl
      AND call.variant_id=v.variant_id )
ORDER BY srcfeature_name ASC, fmin ASC
LIMIT 100
```

Raw Chado query to match the optimized version above

```
SELECT gc.variant_id, gc.marker_id, m.name as marker_name, mt.value as marker_type, gc.stock_id,
      s.name as stock_name, g.stock_id as germplasm_id, g.name as germplasm_name, gc.project_id,
      gc.genotype_id,  a.description as allele_call, gc.meta_data, cf.nid
    FROM chado.genotype_call gc
      LEFT JOIN public.chado_feature cf ON cf.feature_id=gc.variant_id
      LEFT JOIN chado.featureloc loc ON loc.feature_id=gc.variant_id
      LEFT JOIN chado.feature src ON src.feature_id=loc.srcfeature_id
      LEFT JOIN chado.feature m ON m.feature_id=gc.marker_id
      LEFT JOIN chado.featureprop mt ON mt.feature_id=m.feature_id AND mt.type_id=4321
      LEFT JOIN chado.stock s ON s.stock_id=gc.stock_id
      LEFT JOIN chado.organism o ON o.organism_id=s.organism_id
      LEFT JOIN chado.stock_relationship sr ON sr.subject_id=s.stock_id AND sr.type_id=3712
      LEFT JOIN chado.stock g ON g.stock_id=sr.object_id
      LEFT JOIN chado.genotype a ON a.genotype_id=gc.genotype_id
    WHERE g.stock_id IN (:germplasm_ids)
      AND ROW(src.name, loc.fmin) BETWEEN ROW(:backbone, :fmin) AND ROW(:backbone,:fmax)
```

Both of the above queries were run on a production database in order to benchmark query execution times. This was done using EXPLAIN ANALYZE [query] which ensures the query is actually executed by the PostgreSQL query planner over the course of a single day with 9 replicates taking at least 4 hours to complete. The data was then plotted below:

```
data.range = Benchmarking.Genotypes[Benchmarking.Genotypes$query == 'Filter by Range', ]
boxplot(data.range$execution.time~data.range$query.target,
        horizontal=TRUE, ylab="Genotype Matrix Filtered by Range", xlab="Execution Time (ms)")
```

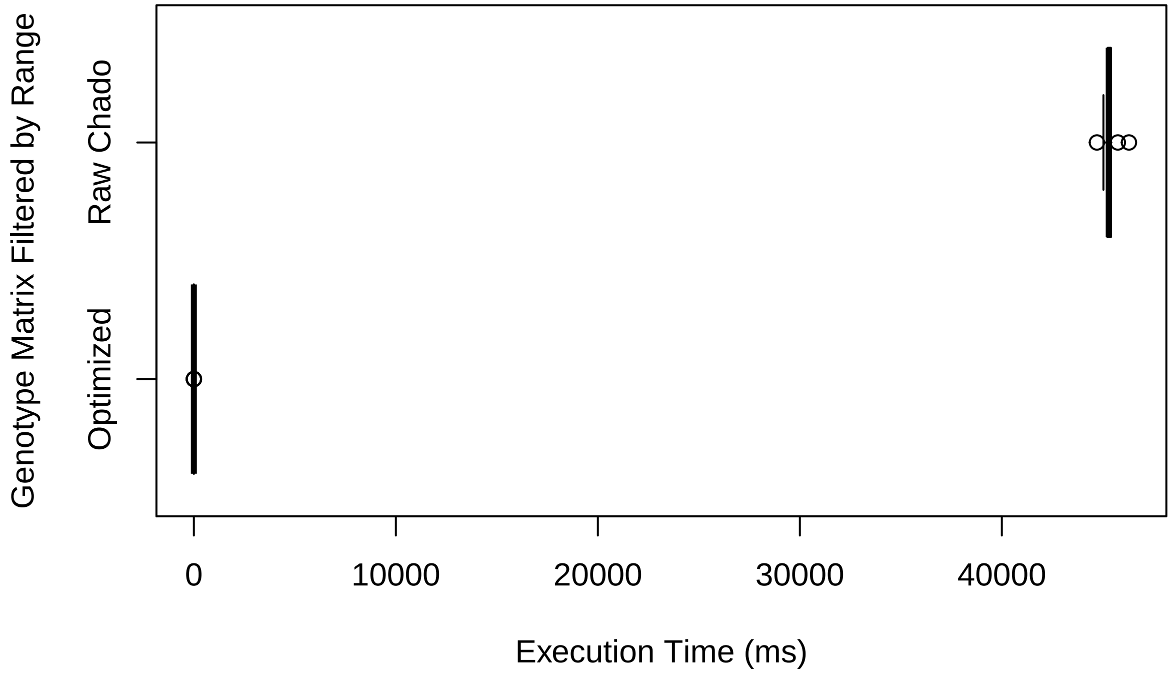

Polymorphic Filter

This is the query executed when you indicate that you would like only variants that are polymorphic returned. Specifically, only variants with different genotype calls between two given germplasm (:poly1 and :poly2) will be shown. Additionally the query includes the same 10 germplasm used for the "Unfiltered Query" (denoted by :germplasm_0 through :germplasm_9 placeholders in the query). Notice that it is much faster to specify that the variant should not be monomorphic then that it should be polymorphic.

The following SQL statements represent accessing the data through the optimized materialized view with indices versus through querying the chado tables directly.

Optimized through Materialized View and Indices

```
SELECT v.*, cf.nid
FROM chado.mview_ndg_lens_variants v
LEFT JOIN public.chado_feature cf ON cf.feature_id=v.variant_id
LEFT JOIN (
    SELECT a.variant_id, a.allele_call=b.allele_call as monomorphic
    FROM mview_ndg_lens_calls a, mview_ndg_lens_calls b
    WHERE
      a.variant_id=b.variant_id
      AND a.germplasm_id=:poly1
      AND b.germplasm_id=:poly2
  ) p ON p.variant_id=v.variant_id
WHERE
  p.monomorphic IS FALSE
  AND EXISTS (
    SELECT true FROM chado.mview_ndg_lens_calls call
    WHERE germplasm_id IN (:germplasm_0, :germplasm_1, :germplasm_2, :germplasm_3, :germplasm_4, :germpl
      AND call.variant_id=v.variant_id )
ORDER BY srcfeature_name ASC, fmin ASC
LIMIT 100
```

Raw Chado query to match the optimized version above

```
SELECT gc.variant_id, gc.marker_id, m.name as marker_name, mt.value as marker_type, gc.stock_id,
      s.name as stock_name, g.stock_id as germplasm_id, g.name as germplasm_name, gc.project_id,
    FROM chado.genotype_call gc
      LEFT JOIN public.chado_feature cf ON cf.feature_id=gc.variant_id
      LEFT JOIN chado.feature m ON m.feature_id=gc.marker_id
      LEFT JOIN chado.featureprop mt ON mt.feature_id=m.feature_id AND mt.type_id=4321
      LEFT JOIN chado.stock s ON s.stock_id=gc.stock_id
      LEFT JOIN chado.organism o ON o.organism_id=s.organism_id
      LEFT JOIN chado.stock_relationship sr ON sr.subject_id=s.stock_id AND sr.type_id=3712
      LEFT JOIN chado.stock g ON g.stock_id=sr.object_id
      LEFT JOIN chado.genotype a ON a.genotype_id=gc.genotype_id
      LEFT JOIN (
        SELECT a.variant_id, ga.description=gb.description as monomorphic
    FROM chado.genotype_call a
    LEFT JOIN chado.genotype_call b ON a.variant_id=b.variant_id
        LEFT JOIN chado.genotype ga ON a.genotype_id=ga.genotype_id
        LEFT JOIN chado.genotype gb ON b.genotype_id=gb.genotype_id
        LEFT JOIN chado.stock_relationship sra ON sra.subject_id=a.stock_id AND sra.type_id=3712
    LEFT JOIN chado.stock_relationship srb ON srb.subject_id=b.stock_id AND srb.type_id=3712
        WHERE sra.object_id=:poly1 AND srb.object_id=:poly2
      ) p ON p.variant_id=gc.variant_id
    WHERE g.stock_id IN (:germplasm_ids) AND p.monomorphic IS FALSE
```

Both of the above queries were run on a production database in order to benchmark query execution times. This was done using EXPLAIN ANALYZE [query] which ensures the query is actually executed by the PostgreSQL query planner over the course of a single day with 9 replicates taking at least 4 hours to complete. The data was then plotted below:

```
data.poly = Benchmarking.Genotypes[Benchmarking.Genotypes$query == 'Polymorphic Variants', ]
boxplot(data.poly$execution.time~data.poly$query.target,
```

```
       horizontal=TRUE, ylab="Genotype Matrix Polymorphic Variants", xlab="Execution Time (ms)")
```

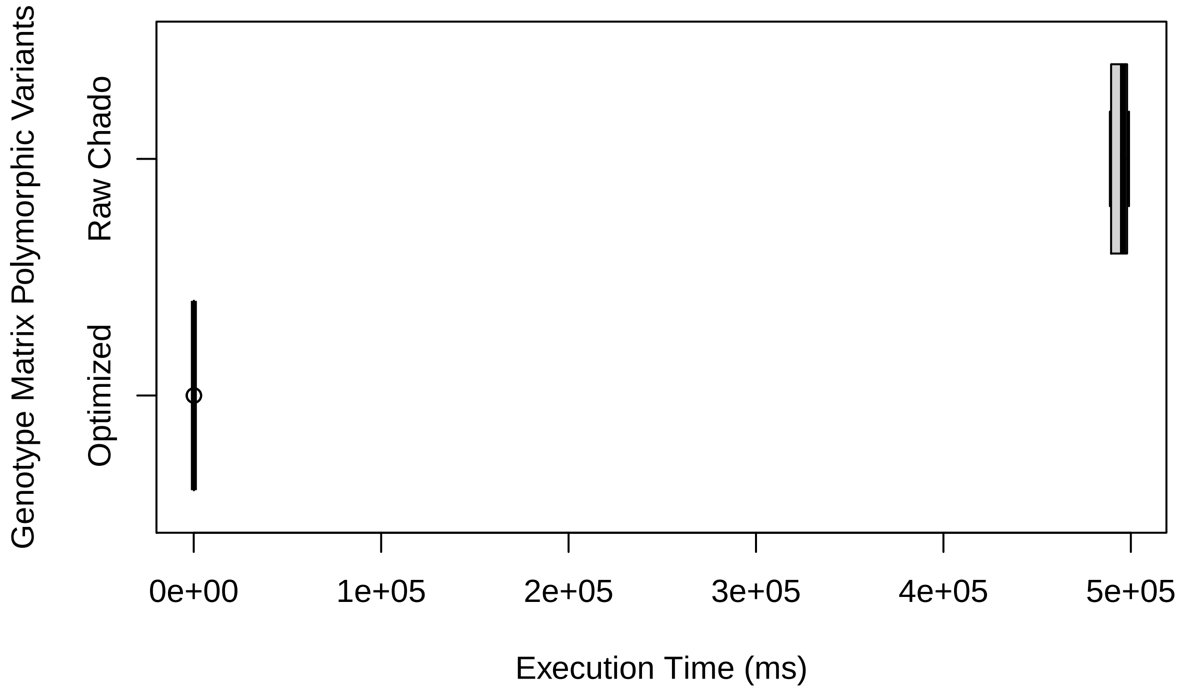

Analyzed Phenotypes

The queries executed represent those used to summarize phenotypic data results. Keep in mind that the results from the queries may be further processed before display and that times reported here do not include render times as stated in the caveats section above.

Quantitative Measurement Distribution

This is the query executed to extract the quantitative data collected for a single trait within a single experiment. The data retrieved represents the values per germplasm and site-year combination for a given trait (denoted :trait_id) and experiment (denoted :project_id). We retrieve the values to ensure support for qualitative traits.

```
SELECT location, year, stock_name, values
FROM chado.mview_phenotype
WHERE experiment_id=:project_id AND trait_id=:trait_id
```

This query is made much simpler thanks to the use of a materialized view. In order to determine the speed increase given by the materialized view, this is the query required to select the same data directly.

```
SELECT
  loc.value as location,
  yr.value as year,
  s.name as stock_name,
  array_to_json(array_agg(p.value)) AS values
FROM chado.phenotype p
  LEFT JOIN chado.cvterm trait ON trait.cvterm_id=p.attr_id
  LEFT JOIN chado.project proj USING(project_id)
  LEFT JOIN chado.stock s USING(stock_id)
  LEFT JOIN chado.organism o ON o.organism_id=s.organism_id
  LEFT JOIN chado.phenotypeprop loc ON loc.phenotype_id=p.phenotype_id
    AND loc.type_id IN (SELECT cvterm_id FROM chado.cvterm WHERE name='Location')
```

```
  LEFT JOIN chado.phenotypeprop yr ON yr.phenotype_id=p.phenotype_id
    AND yr.type_id IN (SELECT cvterm_id FROM chado.cvterm WHERE name='Year')
  WHERE trait.cvterm_id = :trait_id AND proj.project_id = :project_id
GROUP BY trait.cvterm_id, trait.name, proj.project_id, proj.name, loc.value, yr.value, s.stock_id, s.nam
```

Both of the above queries were run on a production database in order to benchmark query execution times. This was done using EXPLAIN ANALYZE [query] which ensures the query is actually executed by the PostgreSQL query planner over the course of a single day with 9 replicates taking at least 4 hours to complete. The data was then plotted below:

```
data.quant = Benchmarking.Phenotypes[Benchmarking.Phenotypes$query == 'Distribution Plot', ]
boxplot(data.quant$execution.time~data.quant$query.target,
        horizontal=TRUE, ylab="Phenotypic Distribution Plot", xlab="Execution Time (ms)")
```

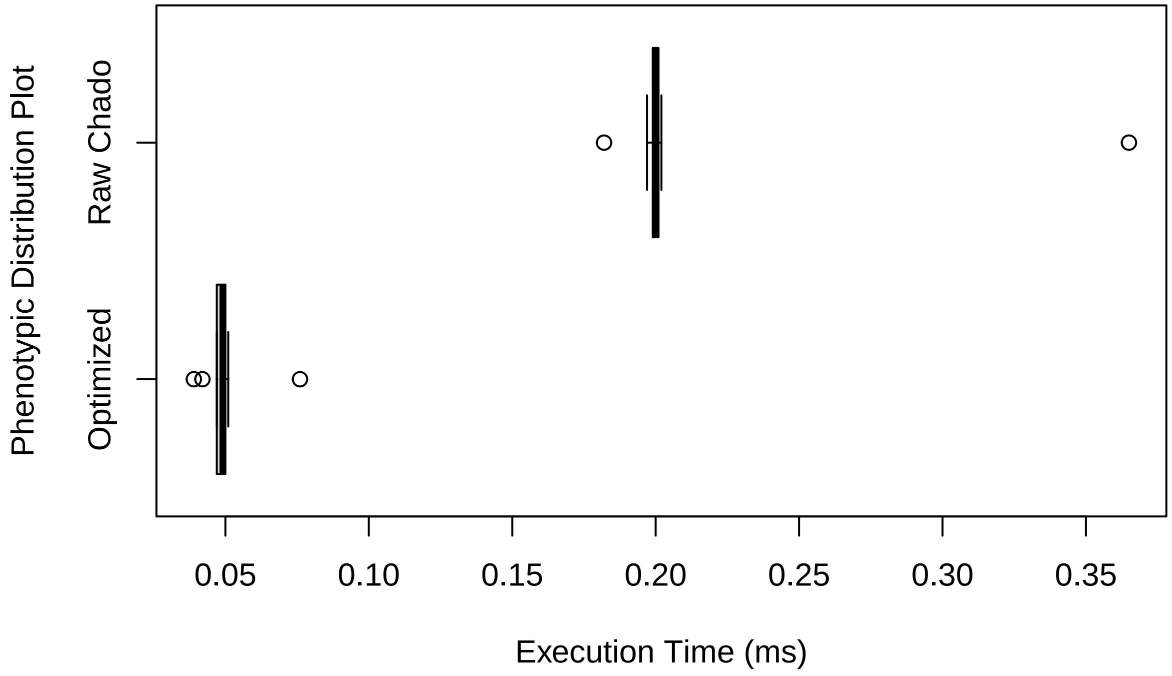

Summary Counts by Genus

This query is executed to summarize the number of phenotypes per genus. It is used to give users an idea of the magnitude of phenotypes available to them for their genus of interest.

```
SELECT * FROM chado.mview_phenotype_summary
```

This query is made much simpler thanks to the use of a materialized view. Since we want the counts for a number of different tables, we use the UNION operator to concatenate the multiple queries into a single one. This is done to fit within the single query per materialized view architecture supported natively by Tripal.

```
(SELECT organism_genus, 1 as num, 1 as count
      FROM chado.mview_phenotype
      GROUP BY organism_genus)
    UNION
      (SELECT organism_genus, 2 as num, count(1)
      FROM (SELECT DISTINCT organism_genus, trait_id FROM chado.mview_phenotype) d2
      GROUP BY organism_genus)
    UNION
      (SELECT organism_genus, 3 as num, count(1)
```

```
      FROM (SELECT DISTINCT organism_genus, experiment_id FROM chado.mview_phenotype) d3
      GROUP BY organism_genus)
   UNION
     (SELECT organism_genus, 4 as num, count(1)
      FROM (SELECT DISTINCT organism_genus, stock_id FROM chado.mview_phenotype) d4
      GROUP BY organism_genus)
   UNION
     (SELECT organism_genus, 5 as num, count(1)
      FROM chado.mview_phenotype
      GROUP BY organism_genus)
```

Both of the above queries were run on a production database in order to benchmark query execution times. This was done using EXPLAIN ANALYZE [query] which ensures the query is actually executed by the PostgreSQL query planner over the course of a single day with 9 replicates taking at least 4 hours to complete. The data was then plotted below:

```
data.summary = Benchmarking.Phenotypes[Benchmarking.Phenotypes$query == 'Summarize Genus', ]
boxplot(data.summary$execution.time~data.summary$query.target,
        horizontal=TRUE, ylab="Phenotypic Summary per Genus", xlab="Execution Time (ms)")
```

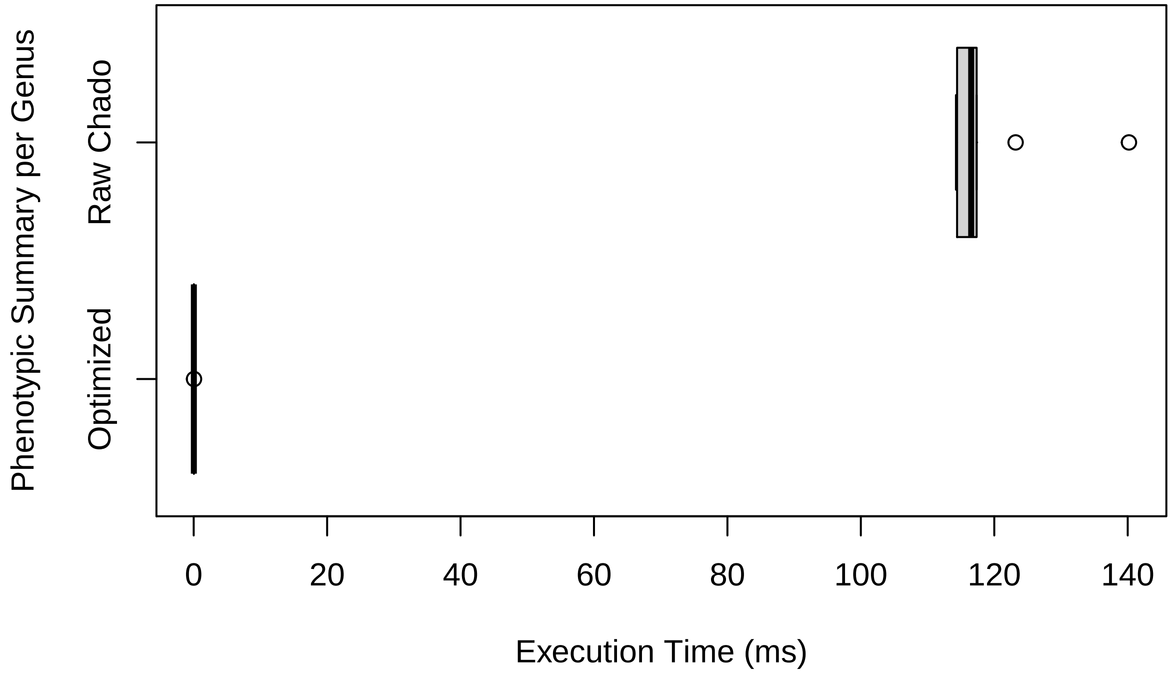

Generating Figure 7

The following demonstrates how Figure 7 was created.

```
par(mfrow=c(3,2))
data.unfiltered = Benchmarking.Genotypes[Benchmarking.Genotypes$query == 'Unfiltered Genotype Matrix', ]
boxplot(data.unfiltered$execution.time~data.unfiltered$query.target,
        horizontal=TRUE, ylab="Unfiltered Genotype Matrix", xlab="Execution Time (ms)")
data.quant = Benchmarking.Phenotypes[Benchmarking.Phenotypes$query == 'Distribution Plot', ]
boxplot(data.quant$execution.time~data.quant$query.target,
        horizontal=TRUE, ylab="Phenotypic Distribution Plot", xlab="Execution Time (ms)")
data.range = Benchmarking.Genotypes[Benchmarking.Genotypes$query == 'Filter by Range', ]
```

```r
boxplot(data.range$execution.time~data.range$query.target,
        horizontal=TRUE, ylab="Genotype Matrix Filtered by Range", xlab="Execution Time (ms)")
data.summary = Benchmarking.Phenotypes[Benchmarking.Phenotypes$query == 'Summarize Genus', ]
boxplot(data.summary$execution.time~data.summary$query.target,
        horizontal=TRUE, ylab="Phenotypic Summary per Genus", xlab="Execution Time (ms)")
data.poly = Benchmarking.Genotypes[Benchmarking.Genotypes$query == 'Polymorphic Variants', ]
boxplot(data.poly$execution.time~data.poly$query.target,
        horizontal=TRUE, ylab="Genotype Matrix Polymorphic Variants", xlab="Execution Time (ms)")
```

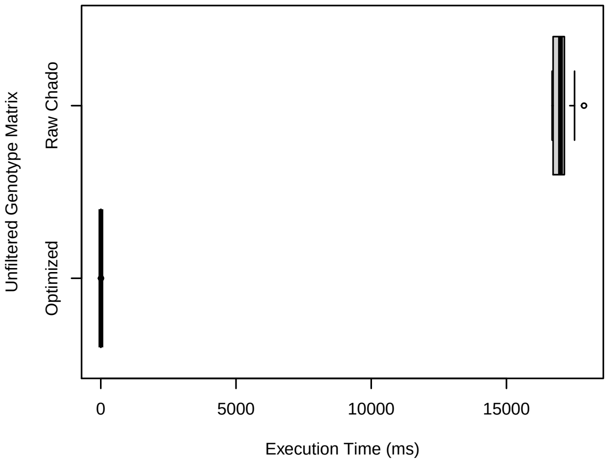
Unfiltered Genotype Matrix
Raw Chado
Optimized
0
5000
10000
15000
Execution Time (ms)

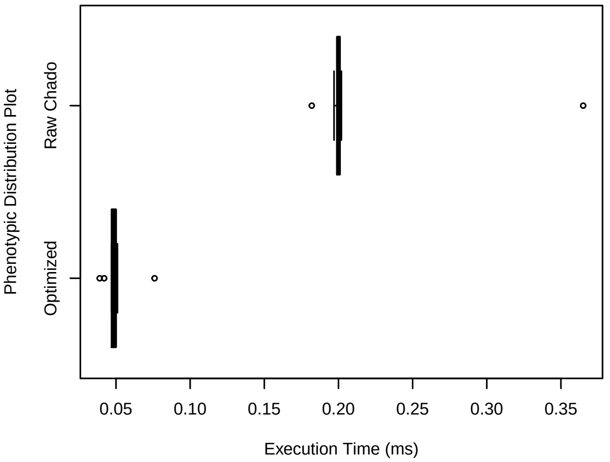
Phenotypic Distribution Plot
Raw Chado
Optimized
0.05
0.10
0.15
0.20
0.25
0.30
0.35
Execution Time (ms)

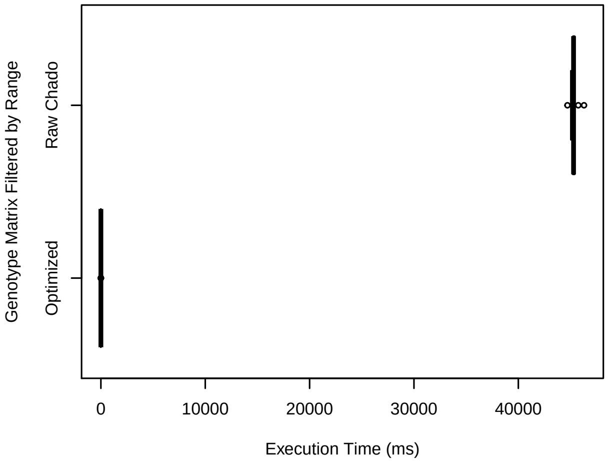
Genotype Matrix Filtered by Range
Raw Chado
Optimized
0
10000
20000
30000
40000
Execution Time (ms)

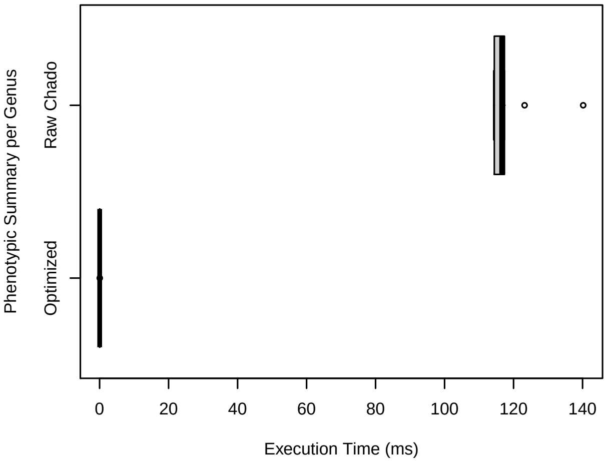
Phenotypic Summary per Genus
Raw Chado
Optimized
0
20
40
60
80
100
120
140
Execution Time (ms)

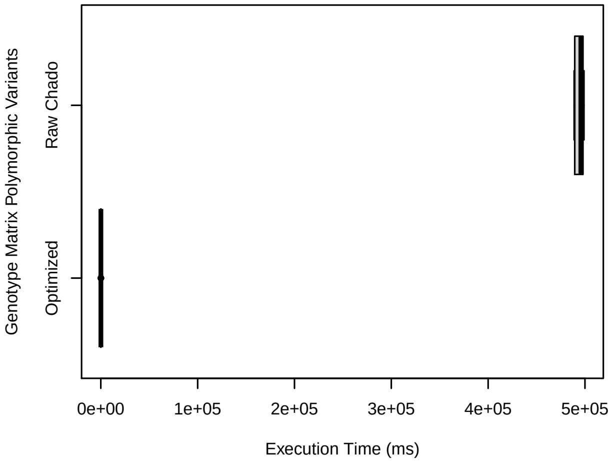
Genotype Matrix Polymorphic Variants
Raw Chado
Optimized
0e+00
1e+05
2e+05
3e+05
4e+05
5e+05
Execution Time (ms)

Automated Timings

Both ND Genotypes and Analyzed Phenotypes have automated timing scripts included in the tests directory of the module. These are provided to facilitate reproducing the benchmarking in this manuscript on your own hardware. There is also a TripalTestSuite Database Seeder provided to allow you to produce your own simulated datasets with similar composition to those used for benchmarking in this manuscript. Instructions for use of these tools has been included in the Contribution, Stress Testing section of the ReadtheDocs documentation for each module.
